# The gut microbiota as a regulator of intestinal stem-cell fate: bacterial and fungal control of differentiation and regeneration

**DOI:** 10.1080/19490976.2026.2734644

**Published:** 2026-09-16

**Authors:** Yetong Shen, Benjamin James Chadwick, Peng Xue, Yiru Gao, Chen Ding

**Affiliations:** a College of Life and Health Sciences, Northeastern University, Shenyang, China; b State Key Laboratory of Mechanism and Quality of Chinese Medicine, Institute of Chinese Medical Sciences, University of Macau, Macau SAR, China; c School of Public Health, Nantong University, Nantong, China; d Department of Pathogenic Microbiology, School of Basic Medical Science, China Medical University, Shenyang, China

**Keywords:** Gut microbiota, intestinal stem cells, intestinal repair, bacterial metabolites, gut mycobiome, epithelial regeneration, host–microbe interaction

## Abstract

Intestinal stem cells (ISCs) sustain epithelial homeostasis and regeneration in a niche that is shaped by epithelial, stromal, immune, and microbial inputs. This review examines how bacterial and fungal signals influence ISC activity and epithelial repair through microbial metabolites, immune relays, and epithelial sensing. Bacterial metabolites and structural ligands act through Wnt/β-catenin, AHR, IL-22–STAT3, Hippo–YAP, and related pathways, and the effects vary according to the ligand involved, responding cell type, and tissue state. Evidence for fungi is more limited: some fungal products engage repair-associated pathways, whereas improved barrier function or reduced inflammation have been observed without directly measuring ISC fate. Therefore, we distinguish direct changes in stem-cell self-renewal, lineage allocation, or regenerative state from indirect evidence based on epithelial repair. This evidence-based framework evaluates how multi-kingdom microbial signals may shape intestinal renewal and identifies key mechanistic gaps.

## 1. Introduction

The microbiota can be considered as an extension of the intestinal stem-cell niche. The intestinal epithelium undergoes constant renewal in response to dietary antigens, commensal microbes, and inflammatory insults.^1^ This regenerative capacity depends on primarily Lgr5^+^ intestinal stem cells at the crypt base, which drive epithelial turnover and give rise to all major differentiated epithelial lineages.^2^ ^,^ ^3^ ISC activity is regulated by a specialized crypt niche that is composed of epithelial cells, mesenchymal populations, and immune-derived signals.

Paneth cells are a key source of epithelial signals in this niche. They secrete niche-supporting mediators such as EGF, Wnt ligands, TGFα, and Notch ligands.^4^ ^,^ ^5^ Nevertheless, host-derived elements do not capture the full regulatory complexity of the ISC niche. Growing evidence indicates that the gut microbiota is an active component of this microenvironment.^6-9^ Gut bacteria and fungal species continuously provide metabolites, structural ligands, and secreted products that can modify epithelial behavior directly or indirectly through niche and immune cells.

### 1.1. ISC fate in homeostasis and repair: Lgr5^+^ stem cells, the crypt niche, and epithelial plasticity

The discovery of Lgr5^+^ crypt base columnar (CBC) cells led to the establishment of the modern framework of intestinal epithelial renewal. Lgr5^+^ intestinal stem cells (ISCs) are located at the crypt base and are the principal actively cycling stem-cell population that continuously sustains epithelial homeostasis. They perform this function by generating absorptive enterocytes and major secretory lineages, including goblet, enteroendocrine, and Paneth cells.^2^ Their transcriptional program is characterized by high expression of *Lgr5*, *Olfm4*, *Ascl2*, and *Tnfrsf19* and is associated with sustained self-renewal and multilineage differentiation.^10-12^ The stem-cell capacity of this population has been demonstrated by organoid assays, in which single Lgr5^+^ cells generated long-lived crypt-villus-like epithelial structures *in vitro*.^4^ Therefore, Lgr5^+^ ISCs are a central cellular engine of epithelial turnover during homeostasis and are an important source of regeneration after tissue injury.

However, Lgr5^+^ ISCs do not function autonomously. Their behavior is determined by the spatially organized crypt niche in which they reside. Signals such as Wnt, R-spondin, and Notch are concentrated near the crypt base, where they maintain stemness and proliferative competence. Daughter cells that are displaced upwards into the transit-amplifying compartment progressively lose exposure to these signals and commit to differentiated lineages.^2^ ^,^ ^4^ ^,^ ^13^ Therefore, intestinal stemness should be viewed not simply as a property defined by Lgr5 expression, but as a functional state that is maintained through continuous communication between epithelial cells and their local microenvironment.

Paneth cells provide a well-characterized example of this niche dependence. Paneth cells are intercalated between Lgr5^+^ CBC cells at the crypt base, they are in direct physical proximity to the active stem-cell compartment, and they provide signals that support ISC survival and expansion. Consistent with this role, co-culture with Paneth cells markedly increases the efficiency of organoid formation by Lgr5^+^ ISCs.^4^ Thus, the crypt niche acts as a spatial signaling hub that concentrates renewal-promoting cues and constrains proliferative activity to the appropriate epithelial compartment. This organization is particularly relevant to microbiota–host interactions because microbial metabolites and inflammation-associated signals can potentially modify epithelial renewal. This process involves acting either directly on ISCs or indirectly by altering neighboring niche cells and the signals that they provide.

Although Lgr5^+^ ISCs dominate epithelial renewal during homeostasis, intestinal repair after injury is substantially more plastic. Regeneration can proceed even when the active Lgr5^+^ compartment is depleted, which indicates that the intestine contains alternative cellular routes that are capable of restoring the stem-cell pool. In some injury conditions, Bmi1-expressing cells increase their progeny output and regenerate Lgr5^+^ ISCs, which supports a context-dependent reserve-like function.^14^ In other models, early transit-amplifying progenitors become a major source of regenerating epithelium after loss of actively cycling ISCs.^15^

Absorptive and secretory progenitors can also reacquire stem-like properties. Following targeted depletion of Lgr5^+^ cells, subsets of these cells re-express ASCL2, undergo dedifferentiation, and contribute to reconstruction of the Lgr5^+^ compartment.^16^ More broadly, differentiated epithelial populations can show considerable regenerative plasticity when the conventional stem-cell niche is disrupted.

Recent single-cell and multi-omics studies have revealed that there is a series of transient regenerative epithelial states that emerge after intestinal injury. Ayyaz et al. identified Clu^+^ revival stem cells that expand during regeneration and contribute to epithelial repair.^17^ More recent studies have shown that these regenerative states can arise from multiple epithelial lineages and involve coordinated TGFβ and Hippo signaling. These findings support a model of reversible cell-state transitions rather than activation of a fixed reserve stem-cell population.

Injury can also induce fetal-like epithelial reprogramming, in which adult epithelial cells transiently reacquire developmental features through pathways that include YAP/TAZ and p53-dependent signaling.^18-20^ Together with injury-associated progenitor and transit-amplifying states identified in organoid and single-cell models,^21^ the findings highlight epithelial plasticity as a central feature of intestinal regeneration.^22^ In this review, we use the term “repair-associated states” to describe these reversible injury-induced epithelial programs and examine how bacterial and fungal signals shape these states through metabolites, secreted peptides, and immune-mediated pathways.

### 1.2. Microbiota as environmental regulators of stem-cell fate

If the classical crypt niche determines where intestinal stemness is maintained, the microbiota helps determine how epithelial cells behave within that niche. Gut microbes are not merely passive residents of the intestinal lumen. Through metabolites, microbial products and host immune intermediates, they provide environmental cues that influence epithelial proliferation, lineage specification, and regenerative responses.^23^ ^,^ ^24^ Therefore, host-derived niche signals establish the structural and signaling framework that is required for epithelial renewal, while microbial inputs dynamically tune the functional output of this system in different physiological and pathological states.

Importantly, microbial regulation is not limited to direct effects on Lgr5⁺ ISCs. Microbial signals can influence multiple levels of the regenerative hierarchy, including the maintenance of actively cycling ISCs, progenitor expansion, lineage commitment, neighboring niche cells, and the induction of repair-associated states after injury. This regulation may occur through direct epithelial sensing or indirectly through immune, stromal, and metabolic intermediates.^24^ Therefore, the microbiota should be viewed as acting on the regenerative system as a whole rather than on a single marker-defined stem-cell population.

A defining feature of these interactions is their context dependence. The biological effect of a microbial cue can vary according to the epithelial state, the type and severity of tissue injury, inflammatory tone, niche composition, and the availability of host-derived signals.^25-29^ Thus, microbial factors should not be classified as simply positive or negative regulators of stem-cell activity. Instead, they act as state-dependent modifiers of epithelial behavior that shift the balance among self-renewal, differentiation, plasticity, and repair according to the surrounding tissue environment.

This perspective also broadens the conventional definition of the intestinal stem-cell niche. Beyond epithelial, stromal, and immune components, the niche is continuously exposed to and shaped by microbiota-derived signals that are sensed and integrated in the local tissue microenvironment. Thus, the microbiota can be considered a dynamic regulatory layer of the intestinal regenerative niche that influences Lgr5⁺ ISC function, alternative regenerative populations, and injury-induced epithelial plasticity^24^ ^,^ ^30^ (Table 1).

**Table 1.** Microbial signals linked to ISC-associated renewal and epithelial repair.

Microbial signal	Representative source	Host pathway	Evidence for ISC involvement	Functional outcome	References
Short-chain fatty acids	Butyrate- and propionate-producing bacteria	HDAC inhibition; GPR41/GPR43; Wnt/β-catenin	ISC-associated—altered proliferation and stem/progenitor markers; effects vary by metabolite and model	Context-dependent control of epithelial renewal	[31,32]
Lactate	*Lactobacillus and Bifidobacterium spp.*	GPR81-dependent Paneth-cell/stromal Wnt3–β-catenin signaling	ISC-associated—GPR81-dependent organoid growth and ISC-associated proliferation were demonstrated	Promotes ISC-associated proliferation and epithelial renewal	[33,34]
Bacterial indoles	Indole-producing commensals	ILC3 AHR–IL-22; epithelial STAT3; Lgr5⁺ ISCs and TA progenitors	Indirect microbial relay with model-dependent downstream IL-22 effects. IL-22 promoted ISC-mediated regeneration in one model but expanded TA cells while depleting Lgr5⁺ ISCs in another	Context-dependent epithelial regeneration; TA-cell expansion does not necessarily indicate expansion of the functional ISC pool	[35–37]
Indole-3-acetic acid	*Acinetobacter radioresistens*	Epithelial AHR; reduced Wnt-associated transcription	Direct—reduced Lgr5-lineage cells and Axin2, Ascl2, c-Myc and Cyclin D1 expression	Slows ISC turnover and early tumor initiation	[38]
Indole-3-ethanol	*Clavispora lusitaniae* P4013B	AHR	Indirect—no ISC-specific readout; responding host cell unresolved	Reduces DSS-induced colitis	[39]
Secondary bile acids	*Clostridium scindens* and other bile acid 7α-dehydroxylating bacteria	DCA-mediated suppression of ileal IL-22	ISC-associated—altered ISC proliferation and secretory differentiation, without lineage tracing	Reduces ISC proliferation and secretory differentiation in the ileum	[40]
Crypt-associated LPS	*Acinetobacter, Delftia* and *Stenotrophomonas*	TLR4-dependent epithelial sensing	Direct—reduced organoid formation and stemness-associated genes while promoting differentiation	Shifts epithelial output toward goblet-cell differentiation	[41]
TLR ligands	LPS, flagellin and LTA	TLR–NOX1–ROS–EGFR	Direct—NOX1-dependent colonic spheroid growth and stem-cell proliferation were demonstrated	Promotes context-dependent NOX1-dependent stem-cell growth	[42]
MDP	Peptidoglycan-producing bacteria	NOD2	Direct—protects Lgr5^+^ ISCs from oxidative stress-induced death	Preserves crypt survival and restitution	[43]
Fungal peptide CD12	*Kazachstania pintolopesii*	ENO1–YAP	ISC-associated—increased stem/progenitor-associated markers and promoted epithelial differentiation; direct ISC fate effects remain unclear	Promotes epithelial repair-associated differentiation	[44]
AMP	*Cladosporium sphaerospermum*	Wnt/β-catenin	ISC-associated—Wnt activity and epithelial proliferation increased, but the directly responding epithelial lineage remains unresolved	Supports epithelial proliferation and repair	[45]
Mucosa-associated fungal communities	Mucosal fungi	CD4⁺ T-cell-derived IL-22	Indirect—improved epithelial protection without measuring ISC fate	Strengthens barrier function after injury	[46]
Amuc_1409	*Akkermansia muciniphila*	E-cadherin/β-catenin dissociation–Wnt signaling	Direct—a defined bacterial protein increased ISC proliferation in organoids and promoted regeneration in mouse intestinal-injury models	Promotes ISC proliferation and injury-associated epithelial regeneration	[47]

### 1.3. Convergent host pathways linking bacterial and fungal signals to epithelial regeneration

Bacteria and fungi generate distinct upstream signals, but many of their effects are interpreted through the same host compartments. These include epithelial stem and progenitor cells, Paneth and stromal niche cells, macrophages, and ILC3/T-cell relays. There are several recurrent pathways at the signaling level, including Wnt/β-catenin, AHR–IL-22–STAT3, macrophage-dependent niche regulation, and direct epithelial regenerative signaling. These shared host routes provide a basis for understanding how taxonomically distinct microorganisms can influence the same regenerative system.

Wnt/β-catenin signaling is one of the clearest points of convergence. Several bacterial taxa have been linked to increased Wnt-associated ISC or niche activity, including *Lactobacillus*, *Bifidobacterium*, and *Akkermansia muciniphila.* ^30^ ^,^ ^31^ ^,^ ^33^ The mucosa-associated fungus *Cladosporium sphaerospermum* engages a similar pathway that promotes Wnt/β-catenin-dependent epithelial repair.^45^

AHR and type 17 immune signaling is another shared route. *Lactobacillus* species can activate AHR-associated ILC3/T-cell responses and promote IL-22-dependent epithelial repair.^35^ *Acinetobacter radioresistens* activates epithelial AHR and constrains Lgr5⁺ lineage expansion.^38^ *Candida albicans* and other mucosa-associated fungi can induce type 17 immune responses, and *Clavispora lusitaniae* has been linked to AHR-dependent mucosal protection.^30^ ^,^ ^46^

Macrophages form a third common host relay. Early-life bacterial communities, including microbiota associated with *Lacticaseibacillus rhamnosus*, support crypt-niche maturation through macrophage-dependent pathways.^48^ Fungi can also act through macrophages but have more variable outcomes. Enteric fungi can support epithelial survival after injury, while wound-associated *Debaryomyces hansenii* promotes inflammatory signaling and delays healing.^49^

Direct epithelial signaling shows greater taxon-specificity. Bacterial species such as *A. muciniphila* and *A. radioresistens* can directly influence epithelial pathways that control proliferation and stem-cell turnover.^38^ ^,^ ^47^ Fungal species such as *K. pintolopesii* and *C. sphaerospermum* also engage epithelial regenerative pathways, particularly YAP- and Wnt-associated signaling.^44^ ^,^ ^45^ These examples suggest that direct epithelial regulation is shared at the level of the target compartment, while the upstream mechanisms are more taxon specific.

These observations support a cross-kingdom view of the intestinal regenerative niche. Bacteria and fungi are distinguished by their upstream signals and taxon-specific mechanisms, but the inputs often converge on a common set of epithelial, stromal, and immune host pathways. The final outcome depends on which host compartment is engaged and on the physiological or inflammatory state of the tissue. The specific microbial molecules and mechanisms that underlie these interactions are discussed in separate sections for bacteria and fungi.

## 2. Bacterial influences on ISC activity and epithelial repair

**Figure 1.**
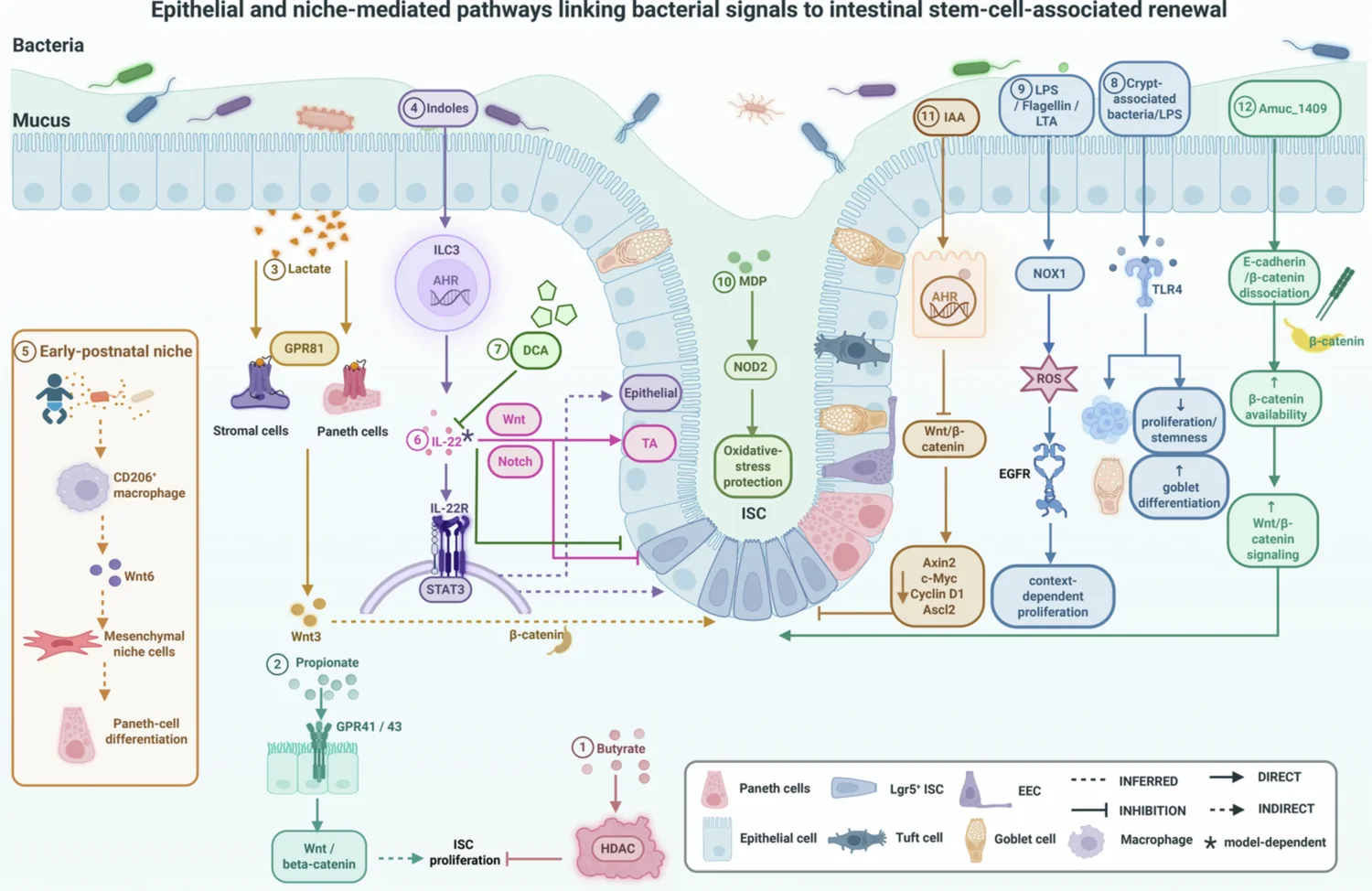
Epithelial and niche-mediated pathways linking bacterial signals to intestinal stem-cell-associated renewal. Bacterial metabolites, structural ligands and secreted proteins regulate intestinal stem-cell (ISC)-associated renewal through direct epithelial sensing and stromal, Paneth-cell, macrophage and immune-cell relays. Circled numbers correspond to the pathway numbers cited in the main text. Butyrate and propionate exert opposing effects through HDAC inhibition and GPR41/GPR43–Wnt/β-catenin signaling, whereas lactate reinforces GPR81–Wnt3-dependent niche support. Indoles activate the ILC3–AHR–IL-22–STAT3 axis, although IL-22 has model-dependent effects on transit-amplifying cells and Lgr5⁺ ISCs. Additional pathways include DCA-mediated IL-22 suppression; early-postnatal microbiota-driven recruitment of CD206⁺ macrophages, which supports Wnt6-dependent mesenchymal niche-cell expansion and Paneth-cell differentiation; TLR4 and NOX1–ROS–EGFR signaling; NOD2-mediated oxidative-stress protection; IAA–epithelial AHR-mediated restriction of Lgr5⁺ lineage expansion; and Amuc_1409-induced β-catenin-dependent Wnt signaling. Solid arrows indicate direct epithelial sensing or experimentally supported molecular steps that do not require an intervening niche-cell relay. Dashed arrows indicate indirect effects mediated through immune cells, macrophages, Paneth cells or stromal niche cells before reaching the ISC-associated regenerative compartment. Dotted arrows indicate inferred relationships, blunt-ended lines indicate inhibition, and asterisks indicate model-dependent effects.

Bacterial-mediated effects on ISC biology are mediated through several interconnected routes, including microbial metabolites, immune intermediates, and direct epithelial sensing.^33^ ^,^ ^36^ ^,^ ^41^ ^,^ ^43^ These mechanisms frequently overlap, and a single bacterial species or bacterial product may act on multiple host compartments simultaneously. The resulting biological effects depend on the nature of the microbial molecules, the anatomical location, and the prevailing inflammatory and injury state of the tissue (Figure 1 and Table 2).

**Table 2.** Representative bacterial taxa linked to ISC and niche responses.

Bacterial taxon	Major factor	Host pathway	ISC evidence and key limitation	References	
*Lactobacillus amylovorus*	Lactate	GPR81–Wnt/β-catenin	Direct—promotes GPR81-dependent porcine organoid growth and ISC-mediated epithelial proliferation; human relevance remains uncertain	[34]	
*Lacticaseibacillus rhamnosus*	Colonization-associated CD206^+^ macrophage expansion	Wnt6–mesenchymal niche support	Indirect—supports Paneth-cell differentiation and postnatal niche maturation; direct ISC fate was not tested	[48]	
*Bifidobacterium spp.*	Lactate and fermentation products	GPR81-dependent Paneth-cell/stromal Wnt3–β-catenin signaling	ISC-associated—lactate increased ISC activity through Paneth-cell/stromal Wnt3 signaling and stimulated *β*-catenin activity in neighboring Lgr5⁺ ISCs	[33]	
*Akkermansia muciniphila*	Propionate, maternal microbial metabolites and Amuc_1409	GPR41/GPR43–Wnt/β-catenin; mTOR; E-cadherin/β-catenin dissociation	Factor-dependent evidence—direct ISC evidence for Amuc_1409 and ISC-associated evidence for propionate and maternal microbial metabolites, with outcomes varying by microbial product, host state and experimental model	[31,47,50]	
*Acinetobacter radioresistens*	Indole-3-acetic acid	Epithelial AHR	Direct—reduces Lgr5-lineage expansion and ISC turnover; evidence derives from organoids and mouse models	[38]	
*Clostridium scindens*	Secondary bile acid production, including DCA	Suppression of ileal IL-22	ISC-associated—reduced ISC proliferation and secretory differentiation were observed, but direct ISC lineage tracing was not performed	[40,51,52]	
Crypt-associated Gram-negative bacteria	LPS	TLR4	Direct—suppresses organoid formation and stemness-associated genes while promoting goblet-cell differentiation	[41]	
Commensal microbiota (taxa not specified)	LPS, flagellin and LTA	TLR–NOX1–ROS–EGFR	Direct—supports NOX1-dependent colonic stem-cell proliferation; outcome depends on ligand, dose and intestinal region	[42]	
Commensal microbiota (taxa not specified)	MDP	NOD2	Direct—protects Lgr5^+^ ISCs from oxidative stress; increased self-renewal or altered lineage allocation was not shown	[43]	

### 2.1. Microbial metabolites as regulators of ISC activity

Among bacteria-derived signals that influence intestinal stem-cell behavior, the most well-characterized are short-chain fatty acids, lactate, and tryptophan-derived indoles.^33^ ^,^ ^35^ These metabolites arise from primarily bacterial fermentation of dietary substrates or amino-acid metabolism. They can regulate epithelial renewal through direct effects on stem or progenitor cells, as well as through niche and immune intermediates.^31^ ^,^ ^33^ ^,^ ^34^ Their actions are highly context dependent. Rather than uniformly promoting epithelial growth, individual metabolites can increase stem-cell activity and regeneration, limit excessive proliferation, or alter lineage commitment. These effects depend on their local concentration, site of exposure, and the physiological or inflammatory state of the intestine.^24^ ^,^ ^30^ ^,^ ^53^

Short-chain fatty acids (SCFAs) predominantly comprise acetate, propionate, and butyrate in the intestinal lumen.^32^ ^,^ ^54^ Butyrate suppresses colonic Lgr5^+^ stem-cell proliferation through inhibition of histone deacetylases (Figure 1, pathway 1), and some studies show an additional role in GPR43-dependent signaling.^32^ ^,^ ^55^ This effect is particularly relevant to the spatial organization of the colon, where differentiated colonocytes normally metabolize butyrate and limit its access to the crypt base.^32^ In contrast, propionate enhances ISC activity in conditions.^31^ Propionic acid engages GPR41 and GPR43, activates Wnt/β-catenin signaling, and stimulates ISC proliferation and epithelial morphogenesis^31^ (Figure 1, pathway 2). Individual SCFAs appear to establish proliferative crypt conditions in opposing directions, which are determined by receptor engagement, epithelial cell state, and local metabolite concentration.

Lactate is involved in another example of metabolite-mediated niche regulation. In addition to being a metabolic nutrient, bacterial lactate can also act as a signaling molecule in the crypt microenvironment. Lactate secreted by commensal *Lactobacillus* and *Bifidobacterium* increases ISC activity through a GPR81-dependent signaling axis.^33^ ^,^ ^34^ Paneth and stromal cells respond to lactate by increasing Wnt3 production, which stimulates *β*-catenin activity in neighboring Lgr5^+^ ISCs^33^ (Figure 1, pathway 3). The result is increased stem-cell proliferation and robust epithelial renewal. This illustrates how a microbial metabolite can reinforce endogenous niche signaling rather than acting exclusively on the stem-cell compartment.

Tryptophan-derived metabolites also demonstrate the importance of the responding cell type. Bacterial indoles can activate AHR in ILC3s, leading to increased IL-22 production. IL-22 then acts on epithelial cells through STAT3 and supports epithelial survival and regenerative responses^35^ ^,^ ^36^ (Figure 1, pathway 4). This regulatory mode is indirect because immune cells rather than by ISCs initially detect the microbial metabolite. In contrast, indole-3-acetic acid produced by crypt-resident *A. radioresistens* acts through epithelial AHR signaling and limits Lgr5^+^ lineage expansion.^38^ These apparently divergent effects indicate that AHR signaling cannot be assigned a uniformly pro-regenerative or suppressive role. Its biological effect depends on the specific ligand, the responding cell type, and the surrounding niche environment.

*A. muciniphila* provides an example of how the metabolic output of a single bacterial species can influence epithelial renewal through several routes. Its production of propionate has been linked to GPR41/GPR43 activation and Wnt/β-catenin signaling, which promotes ISC proliferation and differentiation.^31^ In a developmental context, metabolites derived maternal *A. muciniphila* such as SCFAs and amino acids have also been linked to mTOR activation in the intestinal stem cells of offspring.^50^ In infection or barrier-injury models, *A. muciniphila* increases Ki67^+^ crypt cells, increases Lyz^+^ Paneth cells, increases organoid growth, and rescues ISC function through moderate activation of Wnt/β-catenin signaling.^47^ However, these effects are clearly context dependent.

In ILC3-deficient mice, increased intestinal galactosylation promotes the expansion of *A. muciniphila* in the colonic mucus, and succinate from this species increases the virulence of *Citrobacter rodentium* and the host’s susceptibility to infection.^56^ Thus, the effects of *A. muciniphila* on epithelial renewal cannot be attributed to a single metabolite or regarded as uniformly beneficial. Instead, they depend on the metabolic output, host immune state, and surrounding microbial context.

### 2.2. Immune relays linking bacteria to the crypt niche

Bacterial regulation of ISCs frequently involves immune cells rather than direct epithelial contact alone.^30^ Macrophages, ILC3s, and T cells respond to bacterial structural components and metabolites and then remodel the crypt niche environment. This process entails changing cytokine secretion profiles, modulating stromal Wnt signaling support, sustaining epithelial viability, and tuning local inflammatory tone.^57-59^

During early postnatal development, gut microbial colonization promotes the increase of CD206^+^ intestinal macrophages. These specialized macrophages secrete canonical Wnt ligands, including Wnt6, which maintain mesenchymal niche cells and support Paneth cell differentiation.^48^ Consistently, antibiotic-induced microbiota depletion reduces CD206^+^ macrophages, weakens mesenchymal niche support, and impairs Paneth cell maturation.^48^ *Lactobacillus* colonization or adoptive transfer of CD206^+^ macrophages can partially restore these defects and alleviate necrotizing enterocolitis-like pathology (Figure 1, pathway 5).^48^ These findings identify a microbiota–macrophage–mesenchymal axis that contributes to crypt-niche maturation during early development. However, it has yet to be established whether this Wnt6-dependent mechanism also operates in the adult intestine or during injury-induced regeneration.

ILC3s act as important immune mediators between specific bacterial taxa and the intestinal epithelium. As mentioned in Section 2.1, *Lactobacillus reuteri* produces tryptophan-derived indole metabolites that activate AHR in ILC3s and promote IL-22 production.^35^ IL-22 then activates epithelial STAT3 signaling and can support ISC-mediated epithelial regeneration^36^ (Figure 1, pathway 4). This bacterium–ILC3–epithelial pathway provides an indirect route through which microbial signals influence the regenerative compartment.

However, IL-22 does not produce a uniform response across epithelial cell populations. In other experimental conditions, IL-22 increases transit-amplifying cell proliferation and reduces Lgr5⁺ ISC survival through inhibition of Wnt and Notch signaling (Figure 1, pathway 6).^37^ Thus, increased crypt proliferation should not necessarily be interpreted as an expansion of the functional ISC pool.

Microbiota-derived secondary bile acids also influence this immune axis. Deoxycholic acid (DCA) is generated through bacterial 7α-dehydroxylation of primary bile acids by gut bacteria such as *Clostridium scindens,* ^51^ suppresses ileal IL-22, and reduces ISC proliferation and secretory differentiation (Figure 1, pathway 7).^40^ ^,^ ^52^ These findings indicate that the effect of ILC3–IL-22 signaling on the ISC niche is highly dependent on the context and is shaped by both the microbial metabolic environment and the epithelial population responding to IL-22.

T lymphocytes provide an adaptive immune route through which gut bacteria may influence ISC behavior. Specific bacterial taxa can shape distinct T-cell programs in the intestinal mucosa. For example, through polysaccharide A (PSA), *Bacteroides fragilis* promotes Foxp3⁺ Treg and IL-10-associated responses,^60^ while segmented filamentous bacteria (SFB) strongly induce intestinal Th17 cells and IL-17/IL-22-associated responses.^61^ T-cell-derived cytokines can regulate the fate of ISCs. IL-17A reduces ISC renewal and promotes differentiation,^62^ while IL-17RA signaling in Lgr5⁺ ISCs induces ATOH1, and favors secretory-lineage commitment and epithelial regeneration after injury.^63^ T cells can also directly regulate ISC fate through αEβ7–E-cadherin-dependent interactions that influence Wnt and Notch signaling.^64^

These observations suggest that bacteria-driven changes in T-cell composition may contribute to epithelial regeneration, particularly during infection, dysbiosis, and chronic inflammation. However, there has not yet been any demonstration in the same experimental system of a direct causal pathway linking specific bacteria such as SFB or *B. fragilis* to ISC-mediated regeneration through T cells. Nevertheless, the available evidence supports the possibility that microbiota-induced T-cell programs indirectly influence the balance between ISC maintenance and differentiation.^62^

### 2.3. Direct epithelial sensing of bacterial products

Some bacterial products are sensed directly by epithelial cells within or adjacent to the stem-cell compartment. These signals include conserved microbial structures that are recognized by epithelial pattern-recognition receptors, as well as species-specific metabolites and secreted proteins.^29^ ^,^ ^38^ ^,^ ^41^ ^,^ ^43^ Depending on the ligand and epithelial context, direct sensing can affect proliferation, lineage commitment, cell survival, and regenerative responses. This process provides a local route through which bacteria can influence crypt behavior without requiring an immune-cell intermediate.

One example involves crypt-associated bacteria in the proximal colon, including *Acinetobacter*, *Delftia*, and *Stenotrophomonas*. Bacterial lysates or purified LPS reduce colony formation and the expression of *Ki67*, *Axin2* and *Ascl2* while increasing *Klf4*, *Alpi*, *Krt20*, and *Muc2.* ^41^ This TLR4-dependent response shifts the epithelial behavior away from proliferation and towards differentiation, and features associated with goblet cells prominently increase (Figure 1, pathway 8). These findings suggest that epithelial recognition of crypt-associated bacterial products can directly limit proliferative activity and affect lineage allocation.

However, TLR-dependent signaling is not consistently anti-proliferative. In colonic spheroid cultures, flagellin, LPS, and lipoteichoic acid induce NOX1 expression, while LPS promotes NOX1-dependent stem-cell growth through ROS–EGFR signaling (Figure 1, pathway 9).^42^ The apparent differences in the effects of LPS between experimental systems indicate that TLR signaling cannot be assigned a fixed inhibitory role in the crypt. Its biological output likely depends on the ligand dose, anatomical region, epithelial state, and experimental context. It is currently unclear whether NOX1-dependent redox signaling also influences lineage commitment or injury-induced regeneration.

Muramyl dipeptide (MDP) provides a distinct example, in which direct bacterial sensing primarily affects stem-cell survival rather than proliferation. MDP is sensed through NOD2 and protects Lgr5⁺ ISCs from oxidative-stress-induced cell death, which preserves the stem-cell pool and facilitates epithelial restitution after injury (Figure 1, pathway 10).^43^ This finding is important because it distinguishes the preservation of existing ISCs from increased self-renewal or altered differentiation.

Direct epithelial regulation also extends beyond established pattern-recognition receptor signaling. The crypt resident *A. radioresistens* inhibits ISC turnover through a species-specific small-molecule mechanism. This bacterium releases indole-3-acetic acid (IAA), which is abundant in its outer membrane vesicles. In organoid and *in-vivo* models, IAA activates epithelial AHR signaling, decreases Lgr5⁺ lineage cells, and downregulates mediators associated with proliferation and stemness, including *Axin2*, *c-Myc*, *Cyclin D1*, and *Ascl2* (Figure 1, pathway 11).^38^ These effects slow ISC turnover and suppress early tumor initiation. Because *A. radioresistens* preferentially localizes to the crypt, this IAA–AHR pathway provides an example of spatially constrained microbial control of epithelial renewal. It also contrasts with the discussed ILC3–AHR–IL-22 pathway, in which microbial signals are first perceived by immune cells before affecting the epithelium.

Defined bacterial proteins can also directly affect epithelial signaling. The commensal *A. muciniphila* secretes Amuc_1409, which interacts with E-cadherin, promotes dissociation of the E-cadherin/β-catenin complex, and thereby increases *β*-catenin availability for downstream Wnt signaling (Figure 1, pathway 12).^47^ Importantly, Amuc_1409 increases ISC proliferation and regeneration in organoids and in mouse models of intestinal injury induced by radiation and chemotherapy. This observation provides direct evidence that a defined bacterial protein can promote epithelial repair through ISC regulation.

Direct epithelial sensing allows bacterial cues to have bidirectional effects on crypt dynamics. Depending on the microbial ligand, receptor pathway, anatomical location, and epithelial state, these signals can limit proliferation, promote stem-cell expansion, preserve ISC survival, or affect lineage output. Therefore, direct microbial sensing should be viewed as one component of the broader regulatory environment that shapes intestinal epithelial renewal and repair.^65^ ^,^ ^66^

## 3. Fungal influences on ISC-associated epithelial repair

Compared with bacteria, the role of fungi in regulating intestinal stem-cell fate is much less defined. Fungi are a smaller component of the gut microbiota and have mainly been studied in the context of inflammation, barrier disruption, and opportunistic infection.^67^ ^,^ ^68^ Nevertheless, recent studies indicate that selected mucosa-associated fungi can influence epithelial renewal and repair through both direct epithelial signaling and immune-mediated mechanisms.^44-46^

**Figure 2.**
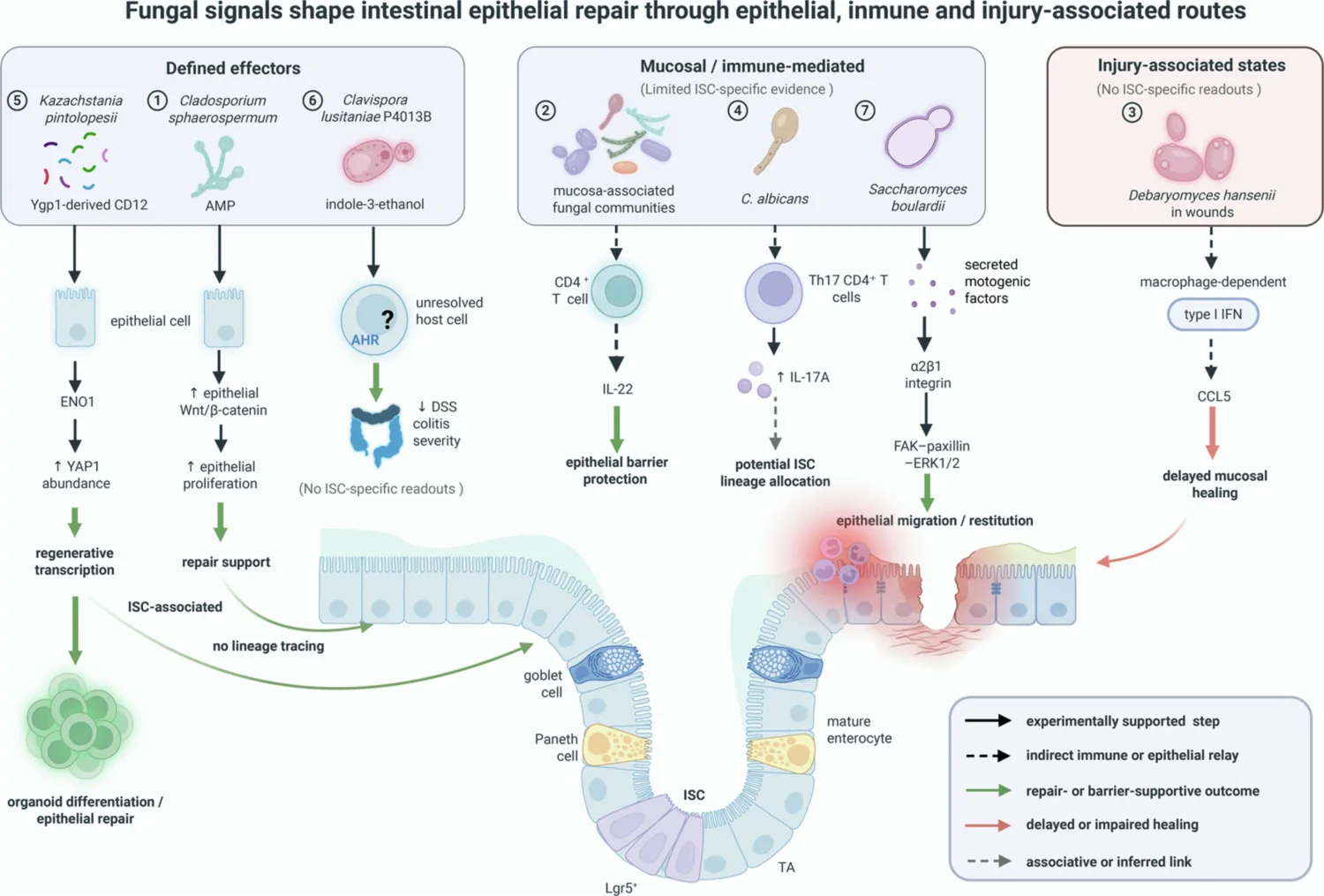
Fungal signals shape intestinal epithelial repair through epithelial, immune and injury-associated routes. Defined fungal effectors and mucosa-associated fungal signals regulate epithelial repair through direct epithelial responses, immune-cell relays and injury-associated macrophage programs. Circled numbers correspond to the pathway numbers cited in the main text. *Cladosporium sphaerospermum*-derived AMP enhances epithelial Wnt/β-catenin signaling, whereas mucosa-associated fungal communities promote CD4⁺ T-cell-derived IL-22 and barrier protection. *Debaryomyces hansenii* delays healing through macrophage-dependent type I IFN–CCL5 signaling. *Candida albicans* induces Th17–IL-17A responses, but its effect on ISC lineage allocation remains inferred. Additional effectors include the *Kazachstania pintolopesii* CD12–ENO1–YAP1 pathway, *Clavispora lusitaniae*-derived indole-3-ethanol–AHR signaling and *Saccharomyces boulardii*-induced α2β1 integrin–FAK–ERK1/2-dependent epithelial migration. Solid black arrows indicate experimentally supported steps, black dashed arrows indicate indirect relays, green and red arrows indicate supportive and impaired-healing outcomes, respectively, and gray dashed arrows indicate inferred links.

Fungal regulation also shows several differences from the better-characterized bacterial mechanisms. Bacterial effects on the ISC niche involve a broad repertoire of metabolites, including short-chain fatty acids, lactate, indoles, and secondary bile acids, as well as pattern-recognition receptor ligands. In comparison, only a limited number of chemically defined fungal signals have been linked to epithelial regeneration. These include secreted peptides, nucleotide metabolites, and tryptophan-derived small molecules.^30^ ^,^ ^44^ ^,^ ^45^ Although some chemical classes overlap between bacteria and fungi, the fungal examples identified have been more strongly associated with individual taxa and their local interaction with the mucosal surface. Accordingly, this section focuses on fungal localization, immune-mediated repair, and defined fungal effectors and distinguishes direct ISC regulation from broader effects on epithelial protection and tissue repair (Figure 2).

### 3.1. From fungal dysbiosis to fungal niche biology

For gut fungi, spatial localization may be particularly important when considering their effects on epithelial repair. Fecal mycobiome profiling does not necessarily capture fungal populations that are abundant at the mucosal surface or in crypt-associated regions, where close proximity to epithelial cells increases the opportunity for local host interactions.^46^ ^,^ ^68^ This spatial feature differs from many bacterial effects, which can be mediated by diffusible metabolites that are produced by a broader range of microbial communities.

Recent studies on mucosa-associated fungi support this view. In individuals with Crohn’s disease, *C. sphaerospermum* is markedly depleted in the ileal mucosa but is relatively unchanged in fecal samples (Figure 2, pathway 1).^45^ The fungus can occupy the intestinal crypt niche and promote epithelial protection, which occurs in part through adenosine 5′-monophosphate (AMP)-dependent increases in Wnt signaling.^45^ Similarly, defined mucosa-associated fungal communities can induce local immune responses that strengthen epithelial barrier function and protect mice from intestinal injury (Figure 2, pathway 2).^46^ These observations shift attention from broad descriptions of “fungal dysbiosis” towards the location, persistence, and functional activity of individual fungal populations at the mucosal interface.

This distinction is also important when evaluating evidence for fungal regulation of ISCs. Most fungal studies have relied on outcomes such as colitis severity, epithelial integrity, or host survival, and direct measurements of ISC fate have been relatively uncommon. Only a small number of fungal models have been used to examine stem-cell-associated markers such as *Lgr5*, *Olfm4*, *Ascl2*, and *Axin2* and particularly functional approaches such as lineage tracing and analysis of lineage allocation.^67^ Therefore, evidence that fungi improve epithelial repair should not automatically be interpreted as evidence for direct regulation of ISC self-renewal or fate.

### 3.2. Immune context and protective and detrimental fungal effects on epithelial repair

Gut fungi can have either protective or detrimental effects on intestinal repair, which depends on the type of fungus, spatial localization, and the immune program they engage.^69-71^ Rather than being intrinsically beneficial or pathogenic, individual fungal populations may produce different outcomes in homeostatic, inflammatory, or injury-associated conditions. Immune-mediated responses are likely to contribute significantly to these context-dependent effects.

In regard to protection, mucosa-associated fungal communities can induce local type-17 immunity and strengthen epithelial barrier function after intestinal injury. Leonardi et al. showed that this protective effect depends at least partly on IL-22 that is produced by CD4⁺ T helper cells (Figure 2, pathway 2).^46^ IL-22 has separately been shown to promote ISC-mediated epithelial regeneration in other experimental settings.^36^ These observations suggest the possibility that fungal induction of CD4⁺ T-cell-derived IL-22 may contribute to epithelial regenerative responses.

Conversely, interactions between fungi and the immune system can also correlate with impaired regeneration. *D. hansenii* preferentially accumulates in incompletely healed intestinal wounds and impairs crypt regeneration and mucosal healing through a macrophage-dependent type-I IFN–CCL5 pathway (Figure 2, pathway 3).^49^ The findings provide relatively direct evidence that a fungal species can affect the local immune environment and compromise epithelial repair.

*C. albicans* also illustrates the context dependence of fungal-induced immunity. Intestinal colonization with commensal *C. albicans* expands fungus-specific Th17 CD4⁺ T cells and increases IL-17-associated responses (Figure 2, pathway 4).^72^ IL-17A can regulate ISC lineage allocation in other experimental settings, which suggests that fungus-induced Th17 responses may potentially influence epithelial regeneration. However, this connection has not been demonstrated directly, and it was unknown whether *C. albicans*-induced IL-17A affects ISC self-renewal or lineage choice in vivo.

### 3.3. Fungal effectors and metabolites that engage regenerative pathways

*K. pintolopesii* is one of the clearest examples of fungi that are involved in regenerative pathways. This species is a murine commensal fungus that promotes epithelial recovery after experimental colitis and chemotherapy-induced intestinal injury (Figure 2, pathway 5).^44^ Colonization by *K. pintolopesii* increases the expression of genes associated with stem-cell activity and epithelial differentiation, including *Lgr5*, *Sox9*, *Hopx*, *Muc2*, and *Chga*. Mechanistically, the fungus secretes Ygp1, which contains a 12-amino-acid peptide, CD12, that can promote epithelial differentiation and accelerate intestinal repair. CD12 binds ENO1 and increases YAP1 protein expression through interaction between ENO1 and *Yap1* mRNA. This process results in the activation of regenerative transcriptional program.^44^

Another example is *C. sphaerospermum*, a mucosa-associated fungus that produces AMP as a bioactive metabolite.^45^ The fungal supernatant and AMP increase epithelial proliferation and Wnt/β-catenin signaling, which is consistent with a role in epithelial regeneration (Figure 2, pathway 1). *C. sphaerospermum* occupies the intestinal crypt niche and provides a direct spatial connection between a defined fungal metabolite and a host pathway that is central to epithelial renewal.^45^ However, it is not completely clear what epithelial population is directly targeted by AMP.

*C. lusitaniae* strain P4013B produces indole-3-ethanol (IEt; tryptophol) through pyruvate decarboxylase-dependent metabolism.^39^ IEt acts as an AHR agonist, and both P4013B colonization and IEt administration improve DSS-induced colitis in an AHR-dependent manner (Figure 2, pathway 6).^39^ This mechanism is particularly interesting in comparison with bacterial AHR signaling because both kingdoms can generate AHR-active metabolites, yet the microbial species, metabolites, and responding host compartments differ. In the case of *C. lusitaniae*, however, the AHR-responsive epithelial population has not been defined, and direct effects on the self-renewal or lineage allocation of ISCs have not been established.

*Saccharomyces boulardii* can also promote epithelial restitution through secreted factors. Its cell-free supernatant increases intestinal epithelial-cell migration through α2β1 integrin–FAK–paxillin–ERK1/2 signaling, but not their proliferation (Figure 2, pathway 7).^73^ The motogenic factors responsible have not yet been molecularly identified, but their characterization may reveal additional fungal-derived mechanisms that support epithelial repair.

These studies demonstrate that fungal products can influence intestinal repair through peptides, purine metabolites, and tryptophan-derived compounds. Nevertheless, the level of ISC-specific evidence varies. Some studies have defined epithelial molecular targets in detail, while others have supported a broader role in epithelial protection or repair (Table 3).

**Table 3.** Fungal regulators linked to ISC-associated epithelial repair: evidence and unresolved questions.

Fungal regulator	Fungal factor	Host pathway	Evidence for ISC involvement	Repair outcome/limitation	References
*Cladosporium sphaerospermum*	AMP	Epithelial Wnt/β-catenin	ISC-associated—increases Wnt activity and epithelial proliferation; directly responding lineage remains unresolved	Supports epithelial proliferation and repair	[45]
*Mucosa-associated fungal communities*	Mucosal colonization signals	CD4⁺ T-cell-derived IL-22	Indirect—improves epithelial protection without measuring ISC self-renewal or lineage choice	Strengthens barrier protection after injury; key fungal members remain unresolved	[46]
*Debaryomyces hansenii*	Wound-associated persistence	Macrophage-dependent type I IFN–CCL5	Indirect—delayed crypt and mucosal healing were demonstrated, but ISC fate was not directly examined	Sustains local inflammation and delays mucosal healing	[49]
*Candida albicans*	Type 17 immune activation	Fungus-specific Th17 CD4⁺ T cells–IL-17A	Indirect/inferred—colonization induces Th17 immunity, but a causal effect on ISC lineage allocation has not been demonstrated	Potential ISC lineage effects remain unresolved	[63,72]
*Kazachstania pintolopesii*	Ygp1-derived CD12	ENO1–YAP1	ISC-associated—organoid differentiation and stem/progenitor-related markers were measured without lineage tracing	Promotes repair after colitis and chemotherapy-associated injury	[44]
*Clavispora lusitaniae* P4013B	Indole-3-ethanol	AHR	Indirect—no ISC-specific readout; responding host cell remains unresolved	Reduces DSS-induced colitis severity	[39]
*Saccharomyces boulardii*	Secreted motogenic factors	α2β1 integrin–FAK/paxillin–ERK signaling	Indirect—epithelial migration and restitution were measured without ISC-specific readouts	Promotes epithelial restitution without increasing proliferation	[73]

Evidence terminology: “Direct” indicates ISC-specific perturbation, lineage tracing, clonal analysis or ISC-resolved functional assays that directly assess self-renewal, lineage output or regenerative capacity. “ISC-associated” indicates changes in stem/progenitor markers, crypt proliferation or non-ISC-resolved organoid outcomes without definitive evidence of ISC fate. “Indirect” indicates niche, barrier, epithelial-survival, inflammatory or tissue-repair outcomes without ISC-specific measurements. “Indirect/inferred” indicates that the demonstrated microbial response is indirect and its proposed connection to ISC fate is inferred from separate experimental evidence. “Associative” indicates taxonomic or community-level correlations without a defined microbial effector or causal host pathway. These terms describe the proximity of the evidence to ISC fate rather than whether microbial sensing occurs directly in epithelial cells or through immune, stromal or other niche-cell relays.

## 4. Cross-kingdom and microenvironmental integration of the regenerative niche

**Figure 3.**
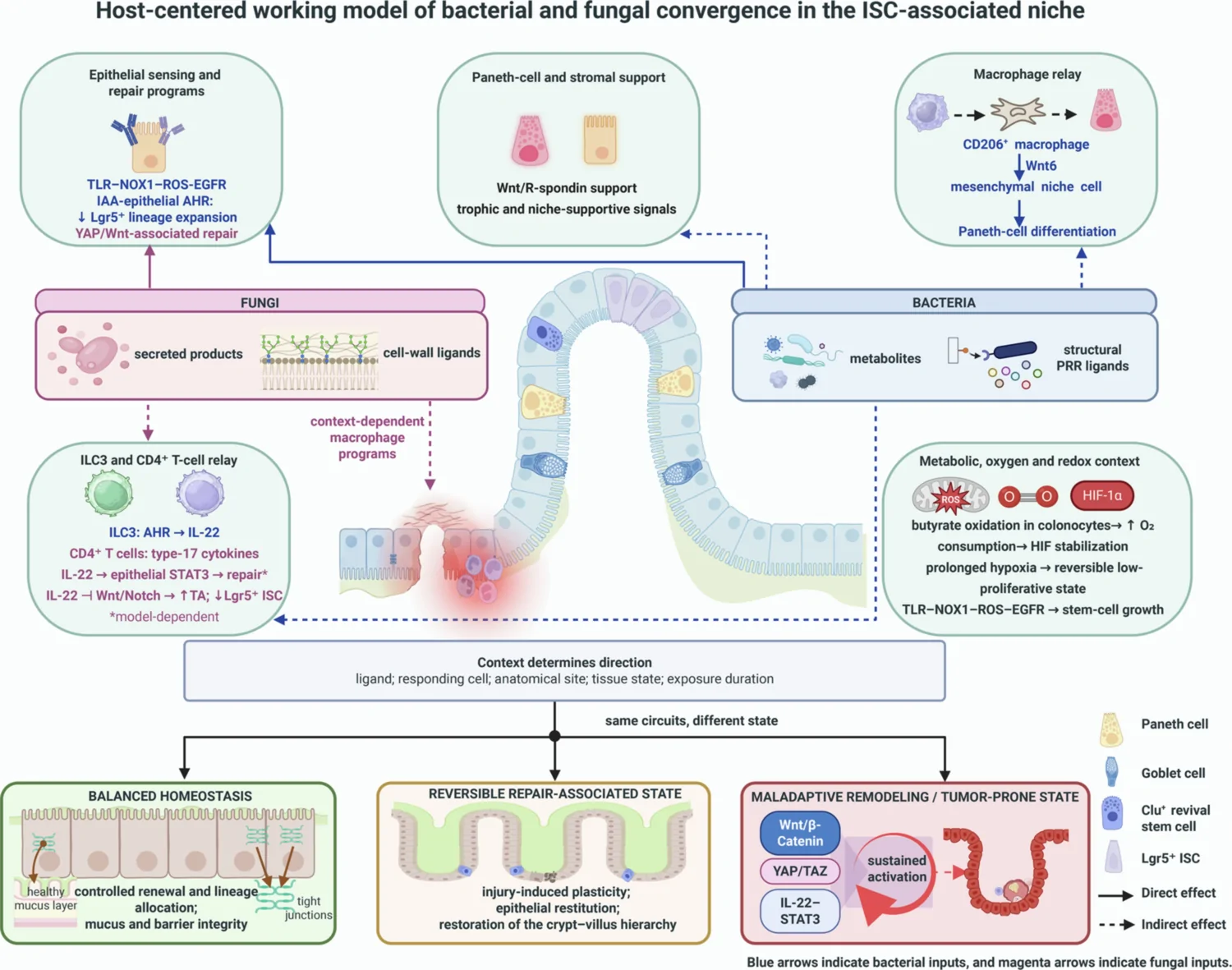
Host-centered working model of bacterial and fungal convergence in the intestinal stem-cell-associated niche. Bacterial and fungal signals originate from distinct metabolites, secreted products and structural or pattern-recognition receptor ligands but are integrated through shared host compartments. These include epithelial sensing and repair programs, Paneth-cell and stromal niche support, macrophage-mediated relays, ILC3- and CD4⁺ T-cell-mediated immune relays, and metabolic, oxygen and redox contexts. Representative convergent mechanisms include Wnt/β-catenin, YAP-associated repair, ligand- and cell-dependent AHR signaling, IL-22–STAT3 signaling, TLR–NOX1–ROS–EGFR signaling and macrophage-dependent niche regulation. Microbiota-derived butyrate oxidation increases oxygen consumption by differentiated colonocytes and supports HIF stabilization, whereas prolonged hypoxia can induce a reversible low-proliferative state in intestinal stem and progenitor cells. The direction and magnitude of these responses depend on the microbial ligand, responding host cell, anatomical location, tissue state and duration of exposure. Accordingly, the same regenerative circuits may support balanced homeostasis and controlled lineage allocation, enable reversible injury-associated epithelial restitution and plasticity, or contribute to maladaptive remodeling and tumor-prone states when activation becomes sustained or occurs in a susceptible tissue context. Solid arrows indicate direct or experimentally supported effects, whereas dashed arrows indicate indirect, relay-mediated or context-dependent effects.

The previous sections have discussed bacterial and fungal regulation as largely parallel inputs into the intestinal regenerative niche. In vivo, however, these signals are neither generated nor interpreted independently. Cross-kingdom interactions and the metabolic state of the surrounding tissue continuously reshape their abundance, localization, and activity. This section focuses on how microbial ecology and the local microenvironment integrate bacterial and fungal signals before they influence the stem-cell compartment (Figure 3).

### 4.1. Spatial organization and cross-kingdom ecological interactions

The intestinal lumen, mucus layer, crypt opening, and injured mucosal surface are distinct microbial habitats and expose host cells to different concentrations of microbial products. Bulk fecal profiling captures only part of the microbial environment that is relevant to epithelial regeneration. This is particularly important for fungi, for which low fecal abundance may obscure enrichment at mucosal or wound-associated sites. For example, *C. sphaerospermum* can occupy the crypt-associated niche, while *D. hansenii* preferentially accumulates in intestinal wounds that have not completely healed.^45^ ^,^ ^49^ Similar spatial confinement occurs for bacteria, including crypt-associated *A. radioresistens*, which allows locally produced signals to act near the epithelial renewal compartment.^38^

Spatial proximity also enables direct bacterial–fungal interactions. In Crohn’s disease, *Candida tropicalis*, *Escherichia coli*, and *Serratia marcescens* are positively associated and can form mixed-species biofilms, with *S. marcescens* facilitating interspecies aggregation.^74^ Although their direct effects on ISC fate are unknown, such communities may increase microbial persistence and affect the inflammatory and metabolic signals that reach the epithelium.

Recent studies show that cross-kingdom interactions can arise from defined metabolic exchange. *C. albicans* and *Enterococcus faecalis* establish a reciprocal interaction in which fungal citrate supports bacterial metabolism, while *C. albicans* detoxifies bacterially generated formate, which facilitates their coexistence.^75^ In another model, *Cladosporium tenuissimum* constrains *C. albicans* through competition for ornithine, while threonine-producing *B. fragilis* enables *C. albicans* to overcome this limitation and aggravate intestinal inflammation.^76^ These findings indicate that cross-kingdom metabolism can reshape microbial abundance and metabolite availability before host cells are engaged, resulting in changes in the signals that occur in the regenerative niche.

### 4.2. Oxygen, hypoxia-inducible factor, and redox metabolism as an interface between microbiota and regeneration

Cross-kingdom interactions occur in a steep gradient of mucosal oxygen that is dynamically shaped by both microbial and epithelial metabolism. In a healthy colon, microbiota-derived butyrate is taken up by differentiated colonocytes and oxidized through mitochondrial *β*-oxidation, which increases epithelial oxygen consumption and helps to maintain physiological hypoxia at the mucosal surface. This metabolic state promotes stabilization of hypoxia-inducible factor (HIF) and supports epithelial barrier function.^77^ Consistent with this model, depletion of butyrate-producing bacteria decreases epithelial oxygen consumption and HIF stabilization, which indicates that the microbiota actively contributes to the maintenance of the landscape of colonic oxygen.^77^ ^,^ ^78^

This relationship is not unidirectional. By shaping epithelial oxygen utilization, microbial metabolites can also influence which microorganisms are favored at the mucosal surface. Butyrate activates epithelial PPARγ-dependent oxidative metabolism, which limits oxygen diffusion into the intestinal lumen and the expansion of facultative Enterobacteriaceae.^79^ In addition, butyrate can stabilize HIF through inhibition of HIF prolyl hydroxylases and provides a complementary mechanism through which a bacterial metabolite can influence epithelial adaptation to low oxygen.^80^

Fungal growth can also affect local oxygen availability and influence neighboring microbial populations. For example, biofilms of *C*. *albicans* generate locally hypoxic microenvironments that enable the growth of otherwise oxygen-sensitive anaerobic bacteria, including *B*. *fragilis* and *Clostridium perfringens*.^81^ Thus, oxygen gradients can emerge from not only host metabolism, but also from the spatial organization and metabolic activity of microbial communities.

This process provides an additional mechanism through which bacterial–fungal interactions may be structured at the mucosal surface. These changes in oxygen availability are also relevant to the regenerative compartment. Experimental manipulation of oxygen tension has been shown to affect intestinal epithelial organization and proliferative behavior, while prolonged hypoxia can induce a reversible low-proliferative state place in human intestinal stem and progenitor cells and change their responsiveness to inflammatory cytokines.^82^

Redox signaling adds a related but mechanistically distinct layer to this oxygen-sensitive environment.^83^ ^,^ ^84^ Microbial ligands can induce epithelial NOX1-dependent ROS production, and basal NOX1-derived ROS can potentiate EGFR signaling and promote colonic stem-cell proliferation.^42^ In contrast, excessive oxidative stress can compromise stem-cell survival and regenerative capacity, indicating that the biological effect of ROS depends on its magnitude, duration, and cellular context.^84^ Thus, oxygen tension, HIF activity, and redox metabolism form an interconnected microenvironmental interface through which microbial ecology may influence epithelial regeneration (Figure 3).

### 4.3. When regenerative plasticity is co-opted for cancer stemness

Pathways that support epithelial regeneration are closely linked to cellular plasticity, but this plasticity can become maladaptive when microbial stimulation is persistent or occurs in genetically susceptible tissue. Therefore, regenerative pathways such as Wnt/β-catenin, Notch, and injury-associated stem-cell program can provide a mechanistic bridge between mucosal repair and tumorigenesis.^22^ ^,^ ^85-87^ Importantly, this does not imply that regeneration is oncogenic; rather, the duration and cellular context of pathway activation determine whether epithelial plasticity supports transient repair or contributes to acquisition of tumor-initiating properties (Figure 3).

*Fusobacterium nucleatum* provides a particularly well-characterized example of this transition. Early studies showed that its FadA adhesin binds epithelial E-cadherin and activates *β*-catenin-dependent oncogenic signaling.^88^ Later work identified Annexin A1 as an additional mediator that sustains *F. nucleatum*-associated Wnt/β-catenin activation in colorectal cancer cells.^89^ These findings established that a bacterial signal can exploit pathways that are also central to normal epithelial renewal.

More recent studies have extended this concept from proliferative signaling to cell-fate plasticity and cancer stemness. *F. nucleatum* promotes the self-renewal of colorectal cancer stem cells and can induce non-stem cancer cells to acquire stem-like properties through lipid metabolic remodeling.^90^ In non-CSCs, recruitment and degradation of the cell-fate regulator Numb occur in association with lipid droplets, which result in the activation of Notch signaling and promote the acquisition of stem-like features.^90^ Consistent with direct interaction with the cancer stem-cell compartment, *F. nucleatum* can also adhere to patient-derived colorectal cancer stem cells, which may potentially involve tumor-associated Gal-GalNAc and CEACAM1-dependent interactions.^91^

Most notably, recent evidence in vivo directly connects *F. nucleatum* to an injury-associated regenerative stem-cell state. *F. nucleatum* was found to colonize deep intestinal crypts and activate LY6A⁺ revival stem cells, which drives their hyperproliferation and subsequent conversion into tumor stem cells through a mechanism that is dependent on LY6A–RPS14.^92^ Genetic disruption of epithelial *Ly6a* or *Rps14* markedly reduces this tumor-promoting effect and provides functional evidence that a microbe can co-opt a regenerative stem-cell population during colorectal carcinogenesis. Therefore, increased proliferation or stem-cell activity should not be interpreted automatically as beneficial regeneration. The long-term outcome depends on the duration of microbial stimulation, the regenerative state of the target cell, and the genetic context of the epithelium.

## 5. Challenges and future directions

Despite rapid progress, fundamental questions persist in regard to how the gut microbiota controls intestinal regeneration, which cannot be exhaustively addressed here. We highlight three challenges that may be particularly important for advancing the field. One major challenge is the definition of the causal architecture of microbiota–ISC regulation. Many microbial perturbations improve epithelial repair, yet it is often unclear whether they act directly on ISCs or indirectly through progenitors, immune cells, stromal cells, or changes in the surrounding niche. Establishing causality will require moving beyond proliferation and disease-severity readouts toward cell-resolved perturbation, lineage tracing, and clonal analysis, which should be ideally integrated with single-cell and spatial approaches.^93^ Central goals should be to determine whether a microbial signal promotes repair, which host cell first senses it, and how the interaction affects subsequent cell-fate decisions.

It is also necessary to understand how cross-kingdom ecology is converted into regenerative information. Bacteria and fungi coexist in spatially organized communities where competition, metabolic exchange, and local environmental conditions continuously reshape microbial activity.^74-76^ As discussed, the defined bacterial–fungal interactions can affect fungal expansion, metabolite availability, and inflammatory outcome, yet it is largely unknown whether and how these ecological interactions ultimately change ISC behavior. Studies should move from analyzing individual taxa in isolation toward resolving the dynamic interaction networks of bacteria, fungi, and hosts at crypt, mucosal, and wound-associated sites.

An important unresolved question is what determines the boundary between adaptive regeneration and maladaptive epithelial plasticity. When pathways that normally enable transient repair are sustained or activated in genetically altered tissue, they can contribute to dysplasia and cancer stemness. Therefore, microbial effects should be evaluated over time and in different states of tissue rather than considered as simply pro- or anti-regenerative. Longitudinal human sampling, patient-derived organoids, and increasingly complex intestine-on-chip models may help to define when a microbial signal supports effective repair and when prolonged exposure instead promotes pathological remodeling.^94^ ^,^ ^95^ Addressing these questions could shift the field from cataloging regeneration-associated microorganisms toward establishing predictive principles of microbiota-controlled epithelial fate. Such efforts would help to identify which microbial signals act, where they act, which host cells receive them, and under what conditions their effects are regenerative rather than pathological.

## 6. Translational perspective and conclusions

The therapeutic potential of the gut microbiota is no longer purely conceptual. Microbiota-based products such as REBYOTA and VOWST have received regulatory approval for the prevention of recurrent *Clostridioides difficile* infection, which demonstrates that complex microbial communities can be developed into standardized therapeutic products.^96-98^ Although these advances have not yet translated to regenerative microbiome therapy, they provide an important translational precedent for harnessing microbial functions in human disease.^99^

For epithelial repair, a logical next step is to move beyond broadly altering microbial composition toward controlling defined microbial functions. Selected live strains may be advantageous when persistent colonization or sustained local activity is required, while defined microbial metabolites, peptides, or proteins could provide greater control over the dose, distribution, and mechanism of action. Fungal products such as CD12 and AMP illustrate how individual microbial molecules can engage regenerative pathways without requiring administration of the entire organism.^44^ ^,^ ^45^ Engineered microorganisms are another strategy that could enable localized delivery or production of regenerative molecules in the intestine. Although these approaches are mostly still preclinical, they provide a plausible route for translating mechanistic discoveries into spatially and functionally controlled interventions.

Importantly, the therapeutic value of a microbial regenerative signal cannot be separated from tissue context. Wnt, STAT3, and IL-22 signaling can support epithelial recovery after acute injury, while prolonged activation of regenerative or plasticity programs may be undesirable in chronically inflamed or tumor-prone tissue.^100-103^ Therefore, therapeutic development should address not only which microbial function should be delivered, but also where, when, and for how long it should act. Stratification according to the disease stage, anatomical location, inflammatory status, and neoplastic risk may ultimately be as important as the selection of the microbial intervention.

These considerations point to a spectrum of translational strategies that have different levels of biological complexity and pharmacological control. Complex microbiota products may be most appropriate for restoring disrupted microbial ecosystems, and defined strains may provide more reproducible biological functions. Microbial metabolites and secreted effectors may provide greater dosing precision, and engineered microorganisms could ultimately combine local delivery with programmable activity. The optimal strategy will depend on whether the therapeutic goal is to restore ecological stability, promote epithelial restitution, or directly modulate regenerative cell behavior. Accordingly, safety assessment should extend beyond short-term improvements in barrier function to include persistent pathway activation, abnormal clonal expansion, and tumor-promoting potential.

In conclusion, microbiota ISC regulation should not be viewed as a linear interaction between individual microorganisms and a fixed Lgr5⁺ stem-cell population. Intestinal regeneration emerges from a plastic epithelial system that is embedded within immune and stromal niches and continuously shaped by bacterial, fungal, and metabolic signals. Therefore, the major translational opportunity is not simply identifying “pro-regenerative microbes” but also converting mechanistically defined microbial functions into controllable, context-appropriate interventions that restore tissue repair without promoting maladaptive epithelial plasticity.

## Data Availability

Data sharing is not applicable to this article as no new data were created or analyzed in this study.
